# Supplementary material for: Ferroptosis: A Novel Type of Cell Death in Male Reproduction
Source: Genes (Basel). 2022 Dec 23;14(1):43. doi: 10.3390/genes14010043 (PMC9858973; doi:10.3390/genes14010043)
Supplement: Supplementary file 1 [file genes-14-00043-s001.zip › genes-2040393-supplementary File S1.pdf]

Method

Firstly, we searched literature in the PubMed database using the following keywords, such as "ferroptosis/iron overload" AND "male infertility/male reproduction/testis/sperm". Among extensive literature, we preferred references in recent 5 years related to male reproduction and ferroptosis. Additionally, articles with high quality such as high impact factors were also our selection criteria. In total, ninety-one references were identified.

Secondly, we carefully read these references and summarized them.

Finally, we classified, summarized and finished this review according to "The discovery of ferroptosis", "Iron metabolism and ferroptosis in the male reproductive system", "The Cyst(e)ine/GSH/GPX4 Axis", "Lipid peroxidation", and "The changes of male reproduction associated with ferroptosis". At last, we provided new insights on male reproductive system diseases from the perspective of ferroptosis.
